# Anaerobic Contribution Determined in Free-Swimming: Sensitivity to Maturation Stages and Validity

**DOI:** 10.3389/fspor.2022.760296

**Published:** 2022-05-17

**Authors:** Eduardo Zapaterra Campos, Carlos Augusto Kalva-Filho, Maria Souza Silva, Tarine Botta Arruda, Ronaldo Bucken Gobbi, Fúlvia Barros Manchado-Gobatto, Marcelo Papoti

**Affiliations:** ^1^Graduate Program in Physical Education, Sports Performance Research Nucleus (NIDE), Federal University of Pernambuco, Recife, Brazil; ^2^Study Group in Physiological Sciences and Exercise (GECIFEX), School of Physical Education and Sport of Ribeirão Preto, University of São Paulo, EEFERP-USP, São Paulo, Brazil; ^3^Laboratory of Applied Sport Physiology, School of Applied Sciences, University of Campinas, São Paulo, Brazil

**Keywords:** anaerobic contribution, swimming, accumulated oxygen deficit, maturation, young swimmers

## Abstract

Evaluation of anaerobic contribution is important under swimming settings (training and modification through ages), therefore, it is expected to change during maturation. The accumulated oxygen deficit (AOD) method can be used to determine the contribution of nonoxidative energy during swimming; however, it requires several days of evaluation. An alternative method to estimate anaerobic contribution evaluation (AC_ALT_), which can also be evaluated without snorkel (i.e., free-swimming, AC_FS_), has been proposed; however, these methods have never been compared. Thus, this study (i) analyzed the effect of maturation stage on AC_FS_ during maximal 400 m swimming (*Part I*), and (ii) compared AOD with AC_ALT_ and AC_FS_, determined in a maximal 400 m effort (*Part II*). In *Part I*, 34 swimmers were divided into three groups, according to maturation stages (early-pubertal, middle-pubertal, and pubertal), and subjected to a maximal 400 m free-swimming to determine AC_FS_. In *Part II*, six swimmers were subjected to one 400 m maximal effort, and four submaximal constant efforts. The AOD was determined by the difference between the estimated demand and accumulated oxygen during the entire effort. The AC_ALT_ and AC_FS_ (for *Part I* as well) was assumed as the sum of lactic and alactic anaerobic contributions. AC_FS_ was higher in pubertal (3.8 ± 1.1 L) than early (2.1 ± 0.9 L) and middle pubertal group (2.4 ± 1.1 L). No difference was observed among absolute AOD (3.2 ± 1.3 L), AC_ALT_ (3.2 ± 1.5 L), and AC_FS_ (4.0 ± 0.9 L) (*F* = 3.6; *p* = 0.06). Relative AOD (51.8 ± 12.2 mL·kg^−1^), AC_ALT_ (50.5 ± 14.3 mL·kg^−1^), and AC_FS_ (65.2 ± 8.8 mL·kg^−1^) presented main effect (*F* = 4.49; *p* = 0.04), without posthoc difference. The bias of AOD vs. AC_ALT_ was 0.04 L, and AOD vs. AC_FS_ was −0.74 L. The limits of agreement between AOD and AC_ALT_ were +0.9 L and −0.8 L, and between AOD and AC_FS_ were +0.7 L and −2.7 L. It can be concluded that AC_FS_ determination is a feasible tool to determine anaerobic contribution in young swimmers, and it changes during maturation stages. Also, AC_FS_ might be useful to measure anaerobic contribution in swimmers, especially because it allows greater speeds.

## Introduction

Anaerobic capacity can be defined as the maximal amount of adenosine triphosphate resynthesized *via* anaerobic metabolism (by the whole organism) during a specific mode of short-duration maximal exercise (Green and Dawson, 1993). Although several methods have been proposed, there is still no gold standard method to assess anaerobic capacity (Gastin, 1994). Medbo et al. (1988) proposed the maximal accumulated oxygen deficit (MAOD) method to assess anaerobic capacity, which uses several submaximal efforts to estimate the theoretical energy demand, and one exhaustive supramaximal effort to determinate the real oxygen demand. Thus, MAOD is estimated by the difference between theoretical demand and real oxygen demand during supramaximal effort (Medbo et al., 1988).

Under swimming settings, previous studies estimated MAOD values using a snorkel and valve system in a swimming flume (Ogita et al., 2003). Reis et al. (2010b) overcame limitations of swimming flume using snorkel in a traditional swimming pool, using front crawl (Reis et al., 2010b) and breaststroke styles (Reis et al., 2010a). These authors used four submaximal efforts and maximal efforts at different distances (100–400 m). As fixed-distance effort was performed to estimate the anaerobic capacity (i.e., athletes did not reach exhaustion), the nomenclature used was accumulated oxygen deficit (AOD) instead of MAOD (Reis et al., 2010b). Besides its use in swimming, AOD and/ or, MAOD determination need(s) several submaximal and maximal efforts separated by a satisfactory recovery phase (Noordhof et al., 2010). Thus, the inclusion of this method in a sports training routine, particularly in swimming, becomes unfeasible.

Therefore, Bertuzzi et al. (2010) showed that an alternative method in cycling was effective to estimate MAOD (MAOD_ALT_) through a single supramaximal effort, which increases its applicability in practical settings. This method considers the sum of the fast component of excess oxygen consumption postexercise [i.e., alactic anaerobic metabolism contribution (Ana_ALA_; Margaria et al., 1933; Di Prampero and Margaria, 1968)], and the net lactate accumulation during the effort [i.e., lactic contribution (Ana_LA_); (di Prampero and Ferretti, 1999)]. Subsequently, several other experiments were conducted, demonstrating its reproducibility (Zagatto et al., 2016; Miyagi et al., 2017), capacity of discriminating athletes with different training levels (Zagatto et al., 2017), and responses to different supplementation strategies (Brisola et al., 2015; Milioni et al., 2016; de Poli et al., 2019), becoming, in fact, an alternative method to estimate MAOD (Valenzuela et al., 2020).

Since a single supramaximal effort is used, MAOD_ALT_ is particularly attractive in a training routine. However, unlike sports where the use of face masks does not compromise the results, as in the case of cycling and running, the use of a snorkel during swimming results in some inconveniences. In this context, the use of a snorkel for swimming (i) makes it impossible to perform specific breathing and the turn in front crawl, (ii) limits breathing in breaststroke and butterfly, and (iii) limits performance of the undulatory underwater swimming. Considering these limitations, AOD determined that the use of the snorkel may be underestimated, especially when determined in a traditional swimming pool. Alternatively, the rapid phase of excessive oxygen consumption (i.e., Ana_ALA_) may be determined in a way similar to the backward extrapolation technique (Montpetit et al., 1981; Monteiro et al., 2020), reducing any influence in swimming patterns. For this, immediately after the effort, swimmers breathe in a face mask connected to the gas analyzer. Using this method, together with net lactate accumulation (Ana_LA_)—it is possible to determine anaerobic contribution in free swimming (AC_FS_), as demonstrated previously (Campos et al., 2017a; Andrade et al., 2021).

Despite this important advance regarding the use of AC_FS_, the validity of this method should be tested to estimate the anaerobic contribution. Considering that changes arising from the maturation process, such as the increase in muscle mass (Boisseau and Delamarche, 2000), and the amount and activity of enzymes related to the glycolytic pathway (Inbar and Bar-Or, 1986; Kaczor et al., 2005) that result in an increase of anaerobic fitness (Inbar and Bar-Or, 1986; Falgairette et al., 1991), an increase in AC_FS_ is expected. Moreover, even though AC_FS_ presents a relation to swimming performance (Campos et al., 2017a), it is important to compare these values with previously validated methods (MAOD_ALT_ and MAOD, or AC_ALT_ and AOD, snorkel when estimated in swimming, respectively (Reis et al., 2010b).

Therefore, the present study: (i) analyzed the effect of maturation stage on AC_FS_ during maximal 400 m swimming, and (ii) compared AOD, AC_ALT_, and AC_FS_ determined in maximal swimming effort. The hypothesis was that AC_FS_ would increase through maturation stages, and that AC_FS_ would be higher than AOD and AC_ALT_ due to a greater swimming speed.

## Methods

### Study Design

**Figure 1:**
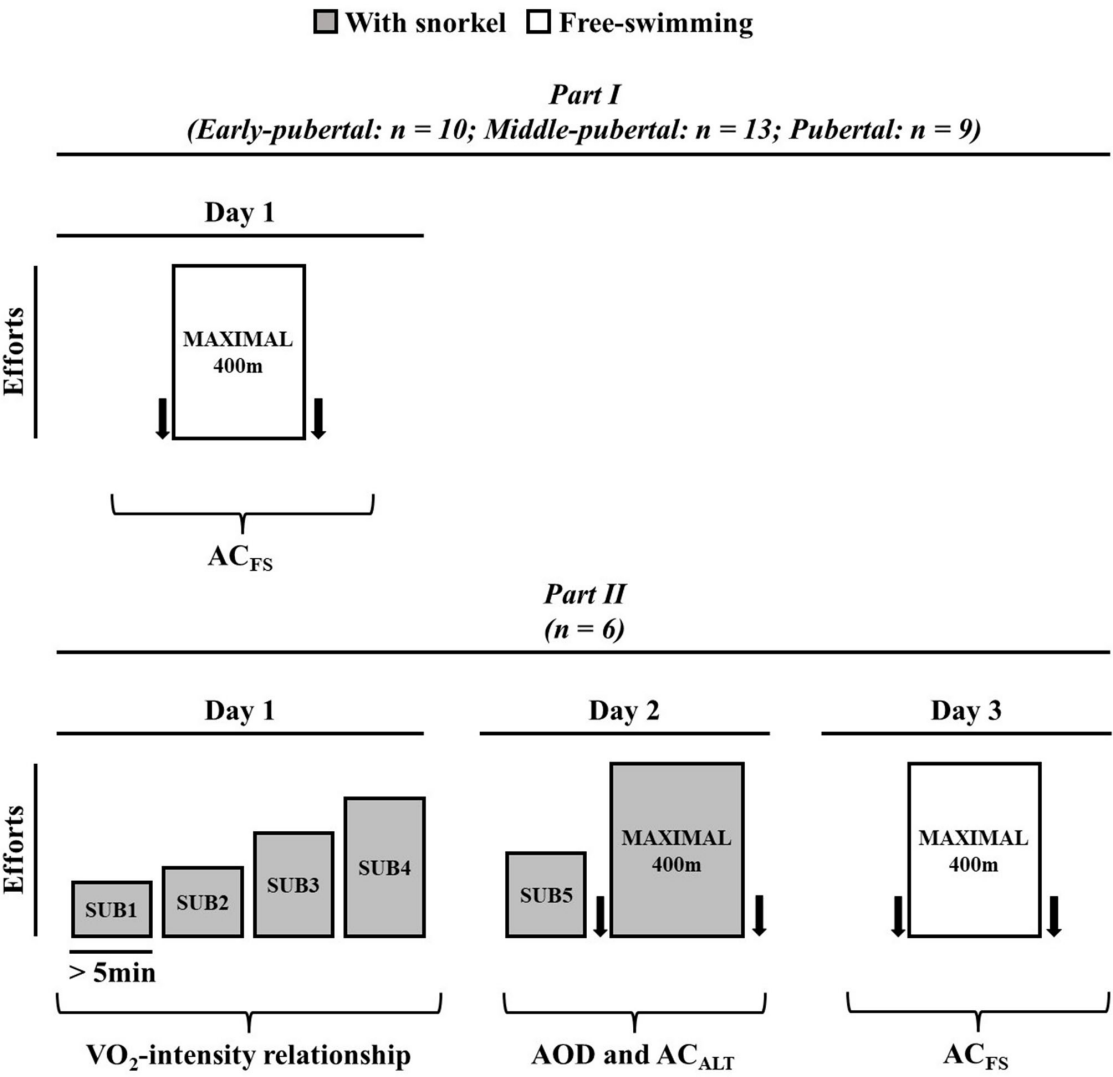
Experimental design for Part I and Part II. Arrows represent blood sample to determine lactate concentration.

In order to determine (i) the modifications of AC_FS_ during maturation stages, and (ii) whether AC_ALT_ and AC_FS_ both determined in a single maximal swimming effort were similar to AOD, the present study was divided into two parts. Figure 1 presents the experimental design of the present study. In *Part I*, swimmers were subjected one maximum front crawl (without snorkel) 400 m effort to determine AC_FS_; and, on the other day, body composition was analyzed by the Dual-energy X-ray absorptiometry (DEXA, General Electric Medical Systems, Fairfield, USA) explained elsewhere (Campos et al., 2012). All tests were performed in a 25-m swimming pool with water temperature of 25 ± 2°C and were preceded by a warm-up of ~1,000 m freestyle swimming of low to moderate intensity determined subjectively by the swimmers. Additionally, swimmers were instructed not to engage in strenuous activity the day before exercise tests and to maintain a consistent routine of training, sleeping, and diet throughout the study.

In *Part II*, swimmers were subjected to three experimental sessions, interspersed by at least 24 h. On the first visit, subjects performed four submaximal efforts aiming to establish VO_2_-speed relationship. On the second day, the subjects were subjected to a submaximal exercise, and a maximal front crawl 400 m effort with snorkel. No warm-up was performed before the tests and the subjects started each trial when their VO_2_ values exhibited two consecutive values within 2 mL·kg^−1^·min^−1^ of that recorded before the first submaximal exercise (observed on the first day; Reis et al., 2010b). This first maximal front crawl 400 m effort (second day trial) was used to evaluate AOD and AC_ALT_ (Figure 1) and the swimmers used snorkel during the effort. After at least 48 h, the swimmers were subjected to another 400 m maximal effort without snorkel (AC_FS_).

### Data Collection and Peak Oxygen Uptake Analysis

Expired gases were collected breath-by-breath using either a gas analyzer (Quark PFT, Cosmed^®^, Rome, Italy) in *Part* I, and a portable gas analyzer (K4b^2^, Cosmed^®^, Rome, Italy) connected to an Aquatrainer snorkel (Cosmed^®^, Rome, Italy) in *Part II*. The gas analyzers were calibrated immediately before and verified after each test using a certified gravimetrically determined gas mixture, while the ventilometer was calibrated preexercise and verified postexercise using a 3-L syringe, in accordance with the manufacturer's instructions. Following the removal of outliers, breath-by-breath data were interpolated to give 1s values (OriginPro 8.0, OriginLab Corporation, Microcal, Massachusetts, USA) to enhance response characteristics of excess postoxygen consumption (EPOC) (Zagatto et al., 2011). Before the maximal 400 m and after 3, 5, and 7 min of recovery, blood samples were collected to determine [La^−^] using a blood lactate analyzer YSI-2300 (Yellow Springs Instruments^®^, OH, USA).

Peak oxygen consumption (VO_2Peak_) was estimated by the backward extrapolation technique, after a maximum front crawl effort of 400 m freestyle, that is, without snorkel. For this, the subjects were instructed to immediately breathe on a face mask (Hans Rudolph, Kansas City, MO, USA) connected to a breath-by-breath gas analyzer system. The equipment was calibrated immediately before the test according to the instruction of the manufacturer. The VO_2Peak_ was obtained using a 30 s backward extrapolation technique (Campos et al., 2017b; Monteiro et al., 2020); for this, VO_2_ values were transformed in logVO_2_, and plotted against time. Through a linear regression the *y*-intercept was considered as VO_2Peak_.

### Subjects

#### Part I

Thirty-four swimmers (19 men, and 15 women) participated in the present study (14.9 ± 2.6 yrs, 58.19 ± 11.88 kg, 161.90 ± 10.98 cm and VO_2Peak_ = 3.30 ± 0.94 L·min^−1^). All the swimmers had at least two years of competitive swimming experience and, had been training an average daily volume of 4,000 m (11–12 yrs), 6,000 m (13–14 yrs), and 8,000 m (>15 yrs), with six trainings·week^−1^ (except 11–12 yrs, that trained 5 times·week^−1^).

#### Part II

Six swimmers (three men and three women) with mean age, height, total body mass, and VO_2Peak_ of 15.1 ± 1.9 yrs, 165.76 ± 8.62 cm, 59.53 ± 11.75 kg, and 3.07 ± 0.57 L·min^−1^ respectively, volunteered to participate in the investigation. All subjects had been swimming training for at least 2 years (average training volume of 7,000 m·day^−1^ and frequency of 5 days·week^−1^).

All procedures were approved by the University's Institutional Review Board for Human Subjects (Human Research Ethics Committee - UNESP - Rio Claro/SP; Ethics Committee Number: 1413/2013), and were conducted according to the Declaration of Helsinki. The athletes and their parents were informed about the experimental procedures and risks and signed an informed consent prior to their participation in the study.

### Procedures

#### Part I

##### Biological Age

Swimmers identified the closest stage representing their body characteristics, using picture boards. Evaluation of pubic hair was done for both genders. Athletes were grouped according to the biological age through the self-assessment method of evaluation of pubic hair proposed by Tanner (1962). This self-rating procedure was previously validated for breast development (B1, B2, B3, B4, and B5) for girls and genitalia (G1, G2, G3, G4, and G5) for boys. Due to the small number of subjects on stages two (*n* = 4) and three (*n* = 6) of this secondary characteristic, the athletes were aggregated into one group. The final groupings were early-pubertal (M2–M3 and G2–G3, *n* = 10), middle-pubertal (M4 and G4, *n* = 14), and pubertal (M5 and G5, *n* = 10).

### Free-Swimming Anaerobic Contribution Determination (AC_FS_)

**Figure 2:**
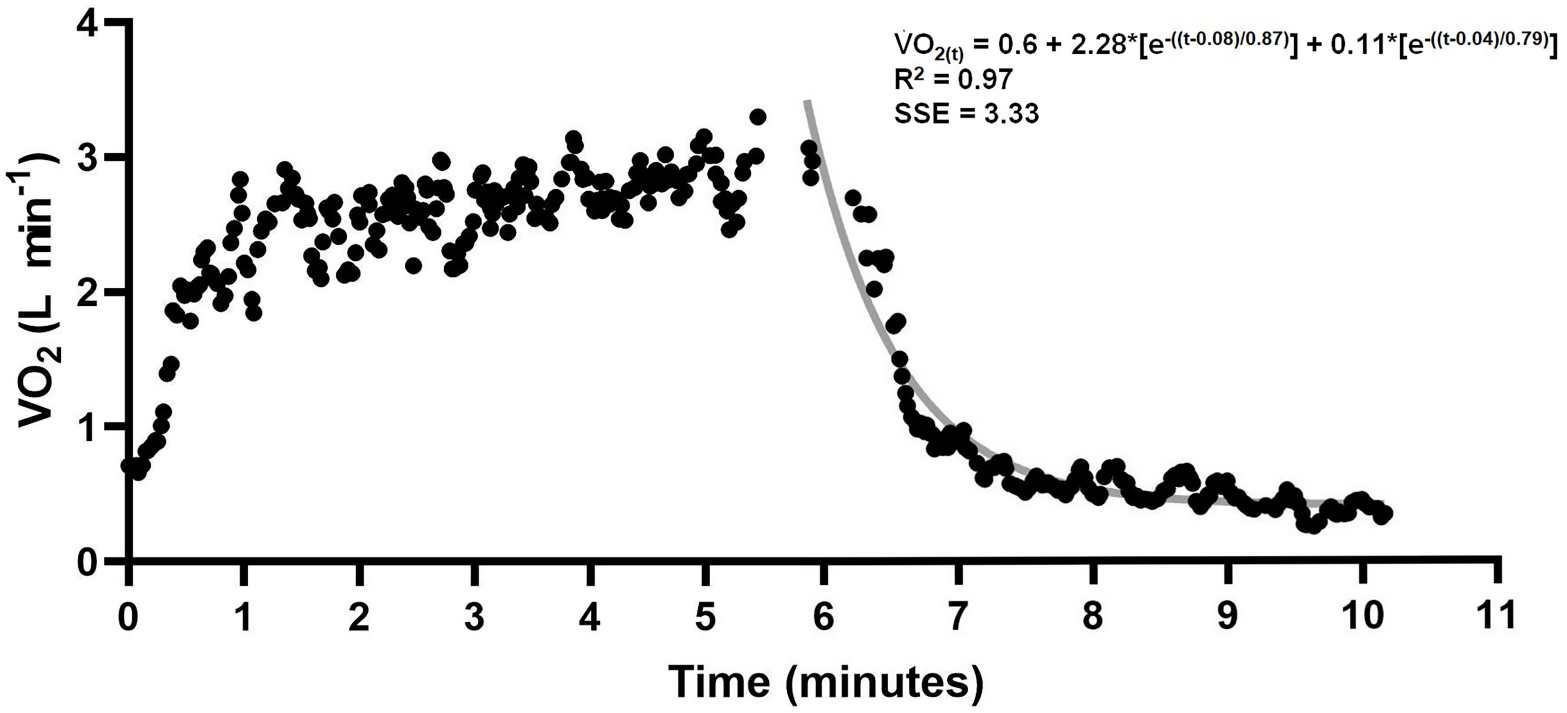
VO_2_ data from 400 m swimming and recovery. Gray line indicates bi-exponential adjustment. Alactic anaerobic contribution was assumed as the product between A1 and t_1_.

Free-swimming anaerobic contribution was determined by the sum of Ana_ALA_ and Ana_LAC_ (Bertuzzi et al., 2010; Zagatto et al., 2011; Kalva-Filho et al., 2015). Swimmers were instructed to immediately breathe on a face mask (Hans Rudolph, Kanss City, MO, USA) connected to a breath-by-breath gas analyzer system (Quark PFT, Cosmed^®^, Rome, Italy) for 5 min (Campos et al., 2017a). The AC_FS_ was calculated in Excel (Microsoft Corporation, Redmond, Washington, USA) and Origin (OriginPro 8.0, OriginLab Corporation, Microcal, Massachusetts, USA). Ana_ALA_ was assumed as the fast component of EPOC. For this EPOC, breath-by-breath measurements obtained during 5 min of recovery were adjusted as a function of time using a bi-exponential model (Equation 1) (Ozyener et al., 2001). The product between amplitude (A_1_) and the fast component time constant (f_1_) was assumed as Ana_ALA_ (Equation 2) (Knuttgen, 1970; Bertuzzi et al., 2010). Ana_LAC_ was obtained by net lactate accumulation (i.e., difference between [La-] peak and baseline values; Δ[La-]), considering a metabolic equivalent of 3 mL·O2·kg-1 for each unit of lactate elevated with maximal effort (di Prampero and Ferretti, 1999). Thus, AC_FS_ was assumed as the sum of Ana_ALA_ and Ana_LAC_ (Equation 3). AC_FS_ values were presented as absolute (L), and relative to body mass (mL·kg^−1^).


(1) $$\begin{array}{rcl} {V\overset{∙}{}O}_{2\left(t\right)} & = & VO_{2BASE}+A_{1}\left[e^{-\left(\left(t-\delta 1\right)/{\text{ƭ}}_{1}\right)}\right]+A_{2}\left[e^{-\left(\left(t-\delta 2\right)/{\text{ƭ}}_{2}\right)}\right] \end{array}$$

(2) $$\begin{array}{rcl} Ana_{ALA} & = & \;A_{1}\cdot \;{\text{ƭ}}_{1} \end{array}$$

(3) $$\begin{array}{rcl} AC_{FS} & = & \;Ana_{ALA}+ANA_{LAC} \end{array}$$

where in Equation 1, VO_2(t)_ is the oxygen uptake at time *t* in recovery time, VO_2BASE_ was the oxygen uptake of at baseline measured before swimming, *A* is the amplitude, δ is the time delay, ƭ_1_ is the time constant (tau) and _1_ and _2_ denote the fast and slow components, respectively. In Equation 2, Ana_ALA_ is the alactic anaerobic contribution and in Equation 3 AC_FS_ is the alternative method to determine anaerobic contribution in a single effort without snorkel and Ana_LAC_ is the lactic contribution. Data of one subject are presented in Figure 2.

#### Part II

##### Conventional Accumulated Oxygen Deficit

Submaximal exercises were performed according to the best 400 m performance of the individual achieved 1 week before the tests (Sousa et al., 2015). The swimmers were instructed to maintain a constant speed during the four submaximal efforts by accompanying sonorous stimuli with markers placed at the bottom of the pool. The distance swam in the submaximal exercises varied from 250 to 400 m. These distances were chosen to ensure a minimal of f5 min of effort, which was related to the VO_2_ plateau attained at 2–3 min (Grassi, 2000). Thus, the mean VO_2_ observed during the final 30 s of the submaximal effort was assumed as the steady-state VO_2_ for the corresponding speed. The linear VO_2_-speed relationship was constructed with the five efforts (four submaximal, and 400 m maximal effort). The mean speed and VO_2_ related to the 400 m maximal effort was also used in the linear regression since this speed is lower than the speed associated with maximal oxygen consumption (≈96%; Reis et al., 2010b).

**Figure 3:**
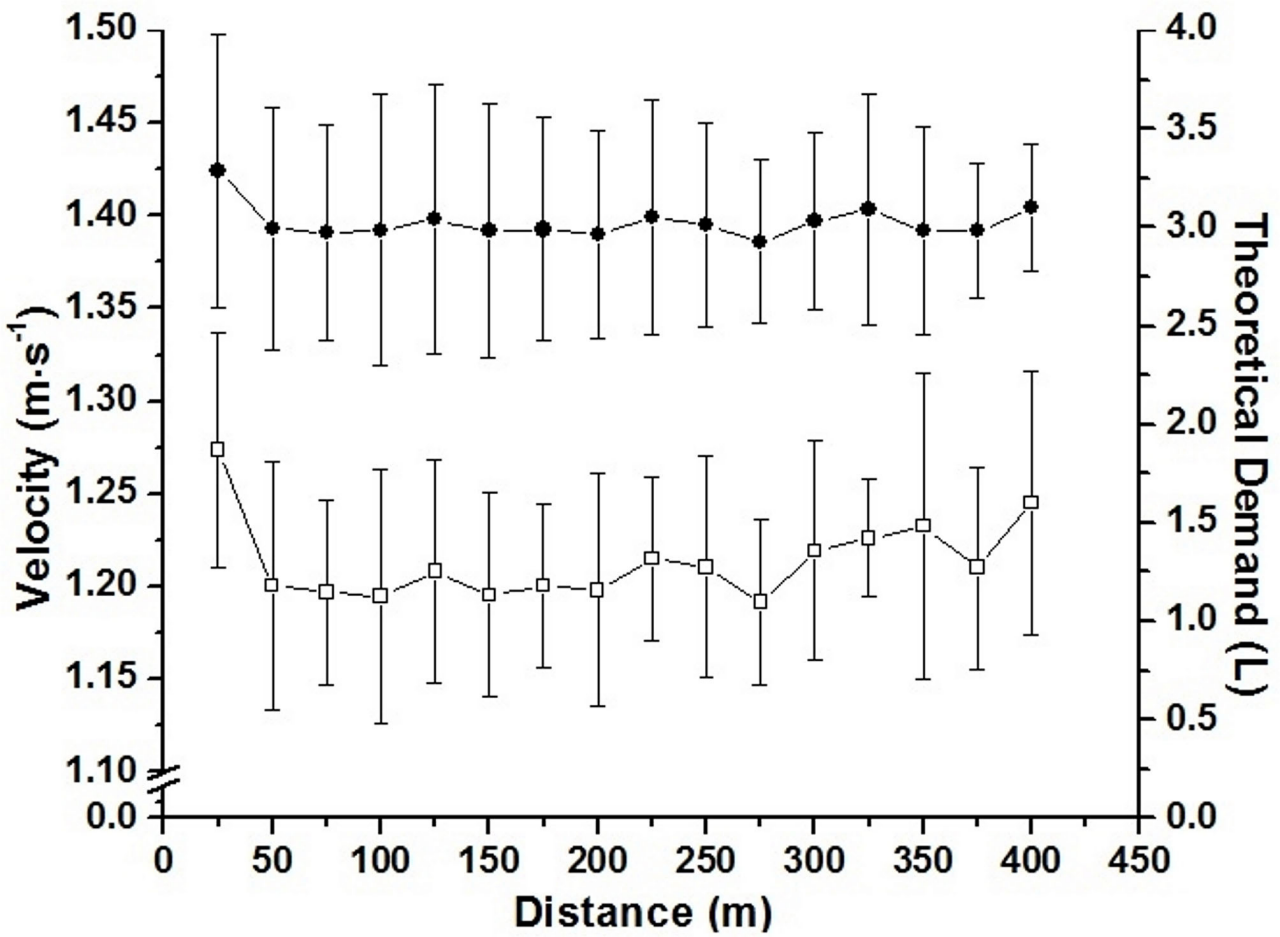
Mean and standard deviation for speed during the 400 m partial (□; left axis), and estimated demand calculated for each 25 m portion (•; right axis).

The accumulated oxygen deficit was assumed as the difference between the estimated demand obtained by VO_2_-speed linear regression extrapolation and the measurement of the VO_2_ during the maximal effort (Medbo et al., 1988). As the swimmers did not use continuous pacing during maximal swimming effort, the estimated demand was calculated for each 25 m (Figure 3). For this, the speed of each 25 m was inserted in the VO_2_-speed linear regression extrapolation, enabling a different estimated demand (i.e., theoretical demand) for each 25 m to be stratified by swimming VO_2_. The difference of the demand for each 25 m and the VO_2_ during the effort was assumed as AOD. AOD was presented in absolute (L), and relative values to body mass (mL·kg^−1^). The AOD calculation was done in Excel (Microsoft Corporation^®^, Redmond, Washington, USA).

### Alternative Anaerobic Contribution (AC_ALT_)

The AC_ALT_ was determined as presented for AC_FS_. The main differences between AC_ALT_ and AC_FS_ are due to the fact that at AC_FS_ the swimmers perform the effort without the snorkel and the fast component of values of EPOC, used to estimate the alactic anaerobic contribution, was obtained immediately after swimming (≈2 seg), while the swimmers swam with snorkel for AC_ALT_.

### Statistical Analyses

Data normality was tested and confirmed by Shapiro–Wilk's test, which permitted the use of parametric tests. Data are presented as mean ± standard deviation (SD). Significance level was set at 5%. The minimal sample size to provide a statistical power of 80% was estimated using G^*^Power software, version 3.1.9.4 (Franz Faul, Christian-Albrechts-Universität Kiel, Kiel, Germany).

#### Part I

The minimal sample size was five participants, considering that the lactic contributions was different between maturation stages during high-intensity efforts, presenting the effect size of 1.798 (Beneke et al., 2007). The comparison between physiological parameters in different biological ages was obtained by one-way ANOVA, and Tukey's posthoc when necessary.

#### Part II

The minimal sample size was six participants, considering that the AOD and AC_ALT_ presented correlations greater than 0.78 (Bertuzzi et al., 2010). ANOVA was used for comparisons between AOD, AC_ALT_, and AC_FS_ repeated measurements. Sphericity was evaluated by Maucly's test, and corrected by Greenhouse–Geisser, when necessary, prior to ANOVA analyses. The Bonferroni's *post-hoc* test was used, when necessary. Moreover, possible correlations and agreements between the methodologies were tested using the Pearson's correlation test, and Bland and Altman (1986) analysis, respectively. Pearson's correlation was also used to test the heteroscedasticity. Correlation coefficients were classified as very small (0.0 – 0.2), small (0.2 – 0.4), moderate (0.4 – 0.7), strong (0.7 – 0.9), and very strong (0.9 – 1.0) (Rowntree, 1981).

For both parts the effect size and confidence interval (90%) of ES was calculated as proposed by Smithson (2001).

## Results

### Part I

The subject's characteristics are presented on Table 1.

**Table 1:** Mean and standard deviation of age, height, weight, total muscle mass (TMM), total body fat (TBF), peak oxygen consumption (VO_2Peak_), baseline lactate concentration ([La^−^]), amplitude of primary component (A_1_), and time constant of primary component (ƭ_1_).

	**Groups**		
	**Early-pubertal**	**Middle-pubertal**	**Pubertal**
	**(*n* = 10)**	**(*n* = 14)**	**(*n* = 10)**
Age (years)	13 ± 2	15 ± 1	18 ± 3
Height (cm)	154.7 ± 10.0	160.6 ± 10.1	170.9 ± 6.9^ab^
Weight (kg)	46.5 ± 9.4	59.5 ± 7.4^a^	68.0 ± 9.5^a^
TMM (kg)	36.9 ± 7.3	46.1 ± 7.6^a^	53.1 ± 8.1^a^
TBF (kg)	9.5 ± 4.6	11.5 ± 5.4	12.1 ± 6.9
VO_2Peak_ (L·min-1)	2.7 ± 0.6	3.3 ± 0.8	3.8 ± 1.1^a^
Baseline [La^−^] (mM)	1.0 ± 0.2	1.6 ± 0.7^a^	1.0 ± 0.4^b^
[La^−^] Peak (mM)	5.5 ± 1.5	7.1 ± 2.4	9.5 ± 3.8^a^
A_1_ (L·min^−1^)	2.2 ± 0.6	2.8 ± 0.8	3.3 ± 1.0^a^
_1_ (sec)	0.6 ± 0.5	0.5 ± 0.2	0.6 ± 0.2

a *Significantly different from early-pubertal group*.

b *Significantly different from middle-pubertal group*.

**Figure 4:**
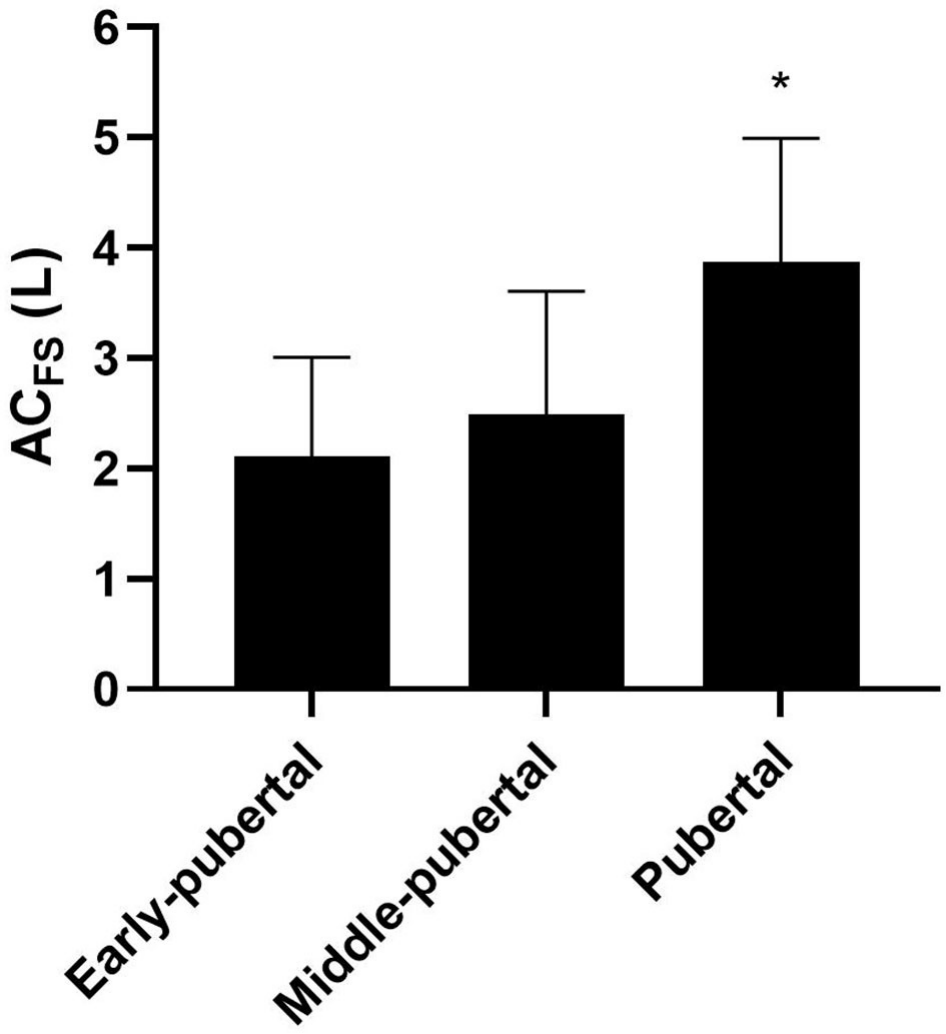
Mean and standard deviation of AC_FS_ (anaerobic contribution) determined in free-swimming in different maturation stages. *Significantly higher than early-pubertal and middle-pubertal.

Figure 4 presents the anaerobic contribution (i.e., AC_FS_) of early-pubertal, middle- pubertal, and pubertal groups determined after the 400 m effort. Absolute Ana_ALA_ only tended to be different among groups [early-pubertal: 1.42 ± 0.84 L; middle-pubertal: 1.47 ± 0.69 L; pubertal: 2.11 ± 0.66 L; *F* = 2.86; *p* = 0.07; Power = 0.52; ηp2 = 0.15; 90% CI (0; 0.30)], without differences in relative Ana_ALA_ [early-pubertal: 30.27 ± 20.70 mL·kg^−1^; middle-pubertal: 24.28 ± 10.13 mL·kg^−1^; and pubertal: 31.63 ± 10.82 mL·kg^−1^; *F* = 0.93; *p* = 0.40; Power = 0.19; ηp2 = 0.05; 90% CI (0; 0.17)]. Pubertal group presented greater absolute Ana_LAC_ than the other groups [early-pubertal: 0.64 ± 0.44 L; middle-pubertal: 1.01 ± 0.51 L; pubertal: 1.75 ± 0.83 L; *F* = 8.72; *p* = 0.001; Power = 0.95; ηp2 = 0.36; 90% CI (0.11; 0.49)], while no differences were found between early-pubertal and middle-pubertal.

Pubertal showed greater relative Ana_LAC_ than early-pubertal [early-pubertal: 12.77 ± 8.42 mL·kg^−1^; middle-pubertal: 16.60 ± 7.24 mL·kg^−1^; and pubertal: 25.44 ± 11.01 mL·kg^−1^; F = 5.49; *p* < 0.01; Power = 0.81; ηp2 = 0.26; 90% CI (0.04; 0.41)]. AC_FS_ were greater in pubertal group than the other groups [early-pubertal: 2.10 ± 0.90 L; middle-pubertal: 2.48 ± 1.12 L; pubertal: 3.87 ± 1.12 L; *F* = 7.79; *p* = 0.002; Power = 0.93; ηp2 = 0.33; 90% CI (0.09; 0.47)], and no differences were found between early-pubertal and middle-pubertal (Figure 4). No differences were found for relative AC_FS_ between groups [early-pubertal: 44.82 ± 19.75 mL · kg^−1^; middle-pubertal: 40.88 ± 15.55 mL · kg^−1^; and pubertal: 57.08 ± 16.49 mL · kg^−1^; *F* = 2.70; *p* = 0.08; Power = 0.49; ηp2 = 0.14; 90% CI (0; 0.29)].

### Part II

Speed ranged between 64.42 ± 0.93 and 80.30 ± 6.85% of 400 m performance in submaximal efforts. The mean time for 400 m was 330.59 ± 13.20 s (mean speed = 1.20 ± 0.04 m·s^−1^) and VO_2Peak_ was 3.07 L·min^−1^. The VO_2_-speed relationship presented values of angular, linear, and determination coefficients of 4.00 ± 1.22 (L·min^−1^)·(m·s^−1^)^−1^, 1.82 ± 1.06 L·min^−1^, and 0.94 ± 0.02, respectively. Figure 3 demonstrates the pacing used by swimmers during the maximal 400 m effort. Table 2 summarizes all parameters related to AOD, AC_ALT_, and AC_FS_.

**Table 2:** Mean ± standard deviation (SD) of accumulated oxygen deficit (AOD), alternative anaerobic contribution (AC_ALT_), and free-swimming anaerobic contribution (AC_FS_) parameters (*n* = 6).

	**Mean**	**SD**
**AOD**		
Estimated demand (L)	13.60	2.79
Accumulated VO_2_ (L)	10.31	1.48
AOD error (L)	1.54	1.25
**AC**_**ALT**_		
Ana_ALA_ (L)	1.36	0.61
Ana_LAC_ (L)	1.87	1.07
Baseline [La^−^] (mM)	1.30	0.27
[La^−^] peak (mM)	10.98	4.07
**AC**_**FS**_		
Ana_ALA_ (L)	1,82	0,30
Ana_LAC_ (L)	2,21	0,79
Baseline [La^−^] (mM)	0.97	0.25
[La^−^] Peak (mM)	12.68	2.29

*Ana_ALA_, alactic anaerobic contribution; Ana_LAC_, lactic anaerobic contribution*.

**Figure 5:**
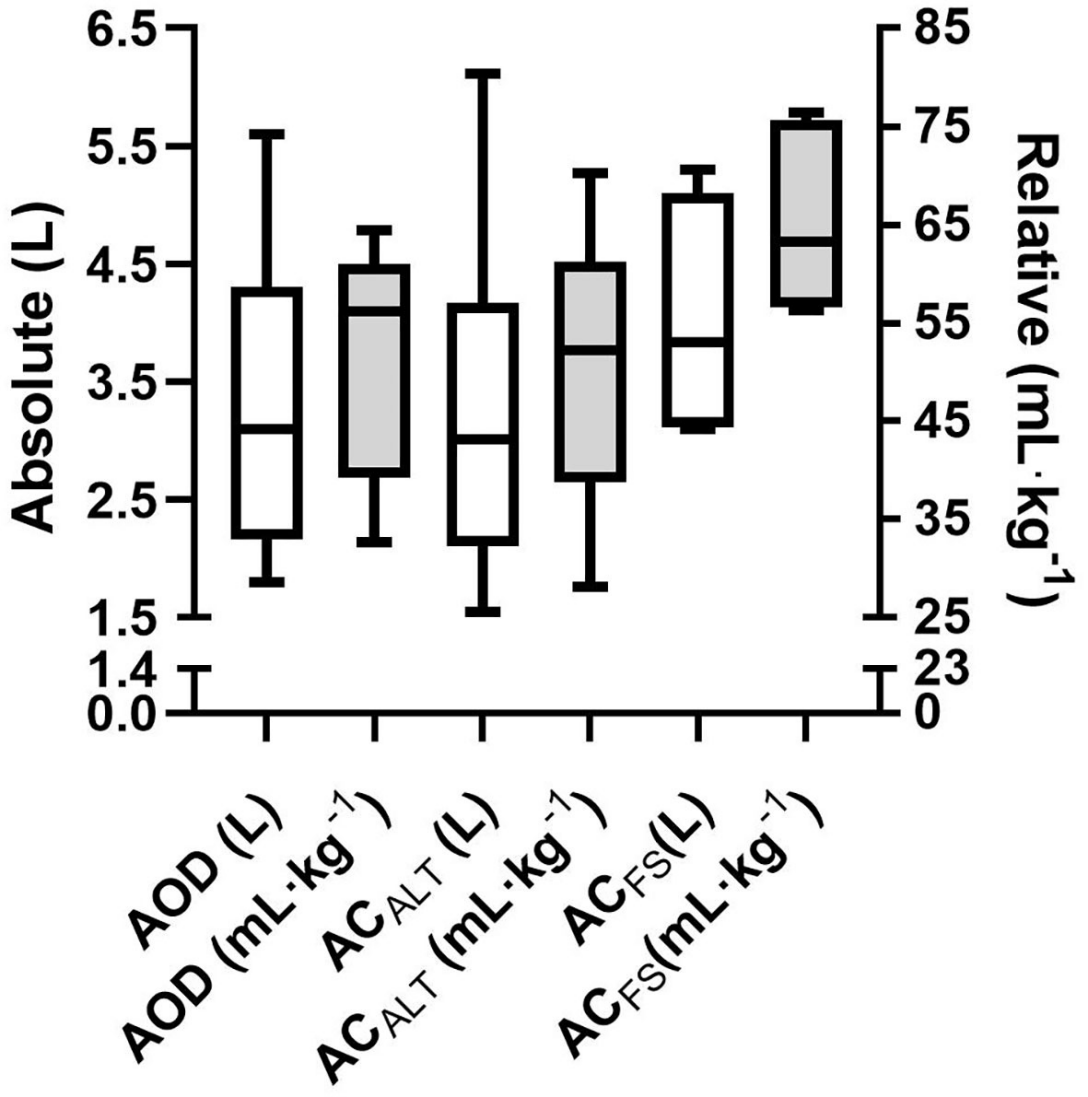
Mean and standard deviation of absolute (white bar) and relative (gray bar) of AOD, AC_ALT_, and AC_FS_.

No differences were found between absolute AOD (3.2 ± 1.3 LO_2_) and AC_ALT_ (3.2 ± 1.5 LO_2_), and AC_FS_ (4.0 ± 0.9 LO_2_) determined in the 400 m maximal effort [*F* = 3.69; *p* = 0.06; Power = 0.54; ηp2 = 0.42; 90% CI (0; 0.60)]. The relative AOD (51.8 ± 12.2 mL · kg^−1^), AC_ALT_ (50.5 ± 14.3 mL · kg^−1^), and AC_FS_ (65.2 ± 8.8 mL · kg^−1^) values presented main effect [*F* = 4.49; *p* = 0.04; Power = 0.62; ηp2 = 0.47; 90% CI (0.01; 0.64)]; however, *post-hoc* analysis did not indicate any differences among values (Figure 5).

**Figure 6:**
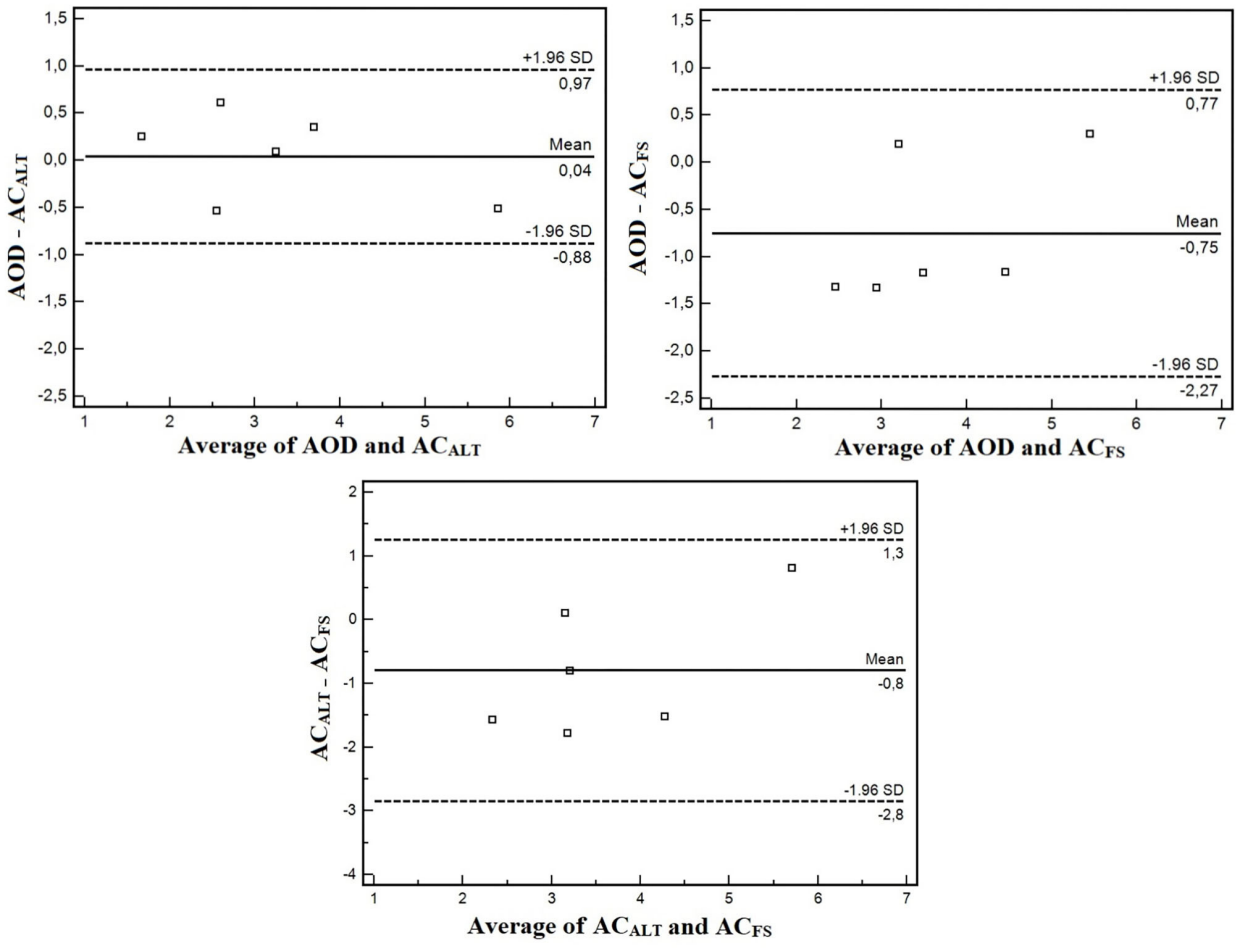
Bland and Altman agreement analysis between AOD and AC_ALT_, AOD and AC_FS_, and AC_ALT_, and AC_FS_.

The agreement analysis between methods are shown in Figure 6. The mean error between AOD and AC_ALT_ was 0.04 L, and between AOD and AC_FS_ was −0.74 L. However, the limits of agreement of AOD and AC_ALT_ were 0.96 and 0.87 L for upper and lower limits of agreement, while between AOD and AC_FS_ were 0.77 L for upper limit and 2.26 L for lower limit (four out of six presented greater AC_FS_ than AOD). AOD was very strongly correlated with AC_ALT_ (*r* = 0.95; *p* = 0.002), and strongly correlated with AC_FS_ (*r* = 0.82; *p* = 0.04).

## Discussion

The aims of the present study were (i) to confirm whether AC_FS_ changes within maturation stages, and (ii) to compare conventional AOD with an alternative method to estimate anaerobic contribution using a single effort with and without snorkel (AC_ALT_ and AC_FS_, respectively). The main findings were that AC_FS_ modifies within maturation stages, and the preliminary validation study did not show differences among AOD, AC_ALT_, and AC_FS_, and that they were strongly correlated (AOD with AC_ALT_: *r* = 0.95; AOD with AC_FS_: *r* = 0.82); however, agreement analysis between AOD and AC_FS_ showed greater lower limits (−2.26 L).

### Part I

In accordance with our hypothesis, AC_FS_ was sensitive to maturation stages in swimmers, with the pubertal group presenting significantly higher absolute AC_FS_ than middle-pubertal and early-pubertal groups. The pubertal and middle-pubertal groups presented greater muscle mass than early-pubertal; however, the difference between middle-pubertal and pubertal was of ≈7 kg on average, which can have practical influence on performance, besides the absence of statistical differences. Thus, expressing AC_FS_ values relative to total body mass and muscle mass is extremely important when comparing the anaerobic indices of swimmers of different biological ages.

These results agree with the findings of Kaczor et al. (2005), which have demonstrated that the quantity and activity of glycolytic enzymes are greater in more mature subjects. The study of Lätt et al. (2009) has also confirmed that net lactate accumulation was significantly greater when swimmers were on Tanner stages 3 and 4 than on stage 2, while no differences were found between stage 3 and 4; however, the authors did not take into account the alactic metabolism. When considering Ana_LAC_ and Ana_ALA_, the latter only tended to be greater (*p* = 0.07) in pubertal than in the other groups. Thus, for swimmers, Ana_LAC_ is the main variable differing between maturation stages. Therefore, the difference in absolute AC_FS_ may be related to Ana_LAC_ since no differences were found in Ana_ALA_ between maturation stages. Furthermore, no differences were detected in relative AC_FS_ between maturation stages, indicating a possible influence of muscle mass on AC_FS_.

Due to its importance in swimming context, a feasible tool to evaluate anaerobic contribution would be important, and AC_FS_ is practical because it enable swimmers to swim freely; however, it was important to compare it with currently used anaerobic contribution determination methods (i.e., AC_ALT_ and AOD).

### Part II

The measurement of energy cost in swimming has received great attention on swimming, since it is important for performance (Zamparo et al., 2000). When calculating the netmetabolic power expenditure, both aerobic and anaerobic contribution must be accounted (Barbosa et al., 2006; Figueiredo et al., 2011). Faina et al. (1997) observed that the time to exhaustion at maximal aerobic speed is closely associated with anaerobic contribution in swimming, highlighting the importance of anaerobic metabolism for maximal efforts. To overcome AOD problems of excessive testing, an alternative method of AOD determination has been proposed using net lactate accumulation and off-transient oxygen consumption (Bertuzzi et al., 2010). As the oxygen consumption can be measured after swimming (Kalva-Filho et al., 2015; Campos et al., 2017a), AC_FS_ would be an even more interesting and applicable tool to evaluate the anaerobic contribution of swimmers without interfering on technique and speed.

The values of AOD observed in the present study were similar to those observed in exhaustive efforts (Ogita et al., 1996), but greater than other investigations that used fixed distance maximal efforts (Reis et al., 2010a,b). Ogita et al. (2003) investigated the possible influence of exercise duration on AOD values obtained in a swimming flume. Those authors observed that anaerobic contribution was similar when exhaustion occurred between one (≈2.8 L) and 5 min (≈2.9 L), with maximal values attained in 2–3 min (≈3.2 L). Thus, maximal AOD values (i.e., anaerobic capacity) can be obtained in a 200 m effort (2–3 min to exhaustion), with no significant differences in relation to a 400 m maximal effort (4–5 min to exhaustion) (Ogita et al., 2003). However, Reis et al. (2010b) observed lower values of AOD in a 400 m than in a 200 or 100 m maximal effort performed in front crawl (≈11.9 mL·kg^−1^, ≈17.5 mL·kg^−1^, and ≈21.0 mL·kg^−1^, respectively). These results were confirmed in breaststroke for 200 and 100 m (≈23.1 mL·kg^−1^ and 22.2 mL·kg^−1^, respectively) (Reis et al., 2010b).

It has been suggested that combining sub and supraanaerobic threshold intensities (i.e., 30–90% of VO_2Max_) affects the precision and validity of the AOD model (Buck and McNaughton, 1999). We did not analyze the anaerobic threshold of swimmers but ensured intensities greater than this physiological index by using the 400 m mean speed as well as a submaximal intensity (i.e., 95% of VO_2PEAK_; unpublished data). Thus, although linear regression is the major concern for AOD calculation, this method is still considered the most acceptable for anaerobic evaluation (Noordhof et al., 2010; Reis et al., 2010b). Different from the present study, the AOD calculation performed in those above-mentioned studies used the effort mean speed to estimate demand, respecting the pace strategy of each swimmer. Thus, we calculated the estimated demand for each 25 m during the maximal effort (Figure 3), increasing the precision of these measurements. This approach together with the five points in the VO_2_-speed relationship, indicate that AOD values were determined in a robust way during the present study, allowing its use to validate AC_ALT_ and AC_FS_.

This is the first study to compare conventional AOD with AC_ALT_ in a maximal swimming effort in swimmers. Bertuzzi et al. (2010) compared a conventional and alternative method, in cicloergometer, to determine anaerobic contribution during an exhaustive cycling effort. Those authors observed similar values, positive significant correlation (*r* = 0.78) and a mean error very close to zero, which agrees with the present findings. Therefore, the difficulties implemented by the need for submaximal exercises to estimate VO_2_-speed relationship are overcome in the alternative method. Finally, determination of AC_ALT_ allows the calculation of Ana_LAT_ and Ana_ALA_ separately, enabling the investigation of different training models on these two metabolisms.

Even though AC_ALT_ decreases the number of evaluations and allows the evaluation of Ana_LAT_ and Ana_ALA_, it was still calculated with swimmers using snorkel during swimming. Besides changes in mechanics during swimming, the apparatus reduces the speed of the swimmers (330.5 ± 13.2 s vs. 303.6 ± 10.8 s), which might limit anaerobic contribution. Another important limitation refers to the impossibility of swimmers performing the turns and the underwater dolphin kick, a technique that has been commonly observed in swimming events. The use of snorkel also limits the use of “filipina” during breaststroke swimming, in addition to being uncomfortable for swimmers, limiting its use in practical settings.

We have shown no differences between AC_FS_ with AC_ALT_ and AOD; however, a tendency was detected in absolute values and an effect was found for relative anaerobic contribution (without detection in posthoc analysis). This might have occurred due to the reduced sample size. It is important to note that the limits of agreement between AOD and AC_FS_ highlighted a lower limit of 2.26 L. Four out of six presented significantly greater AC_FS_ than AOD (mean difference of 1.24 L). Thus, even though no statistical differences were observed, free swimming anaerobic contribution evaluation (AC_FS_) might be recommended because it allows the athletes to perform in greater intensity, which is especially important since swimmers did not reach exhaustion during swimming.

The limitations of the present study were that athletes (both men and women) were evaluated in *Part I* which might have influenced the comparison between maturation stages, and the small sample size in Part II. It would be desirable to confirm these results with a larger sample size. Finally, for the Ana_ALA_ determination, 5 min of recovery was used. Bertuzzi et al. (2016) have observed that a minimum of 6 min is required for Ana_ALA_ evaluation; however, 5 min of recovery have been used in other studies (Kalva-Filho et al., 2015; Campos et al., 2017a; Andrade et al., 2021), and the fast component happens in the 1st min of recovery. Moreover, studies could also use bi-exponential decay equation as proposed by Scheuermann et al. (2011)—since it does not assume that athletes will reach baseline values at the end of recovery—and compare Ana_ALA_ using both Scheuermann et al. (2011) and Ozyener et al. (2001) equations.

## Conclusion

Collectively, it can be concluded that the AC_FS_ is sensitive to maturation stages, and no differences were detected with AOD and AC_ALT_. Therefore, AC_FS_ might be useful to estimate anaerobic contribution in swimmers, facilitating its determination in practical settings, because swimmers are able to swim freely, which increases the speed of swimming.

## Data Availability Statement

The raw data supporting the conclusions of this article will be made available by the authors, without undue reservation.

## Ethics Statement

The studies involving human participants were reviewed and approved by Human Research Ethics Committee - UNESP - Rio Claro/SP; Ethics Committee Number: 1413/2013. Written informed consent to participate in this study was provided by the participants' legal guardian/next of kin.

## Author Contributions

EC, MS, TA, CK-F, and RG collected the data. EC, CK-F, FM-G, and MP wrote the manuscript and delineated the study. All authors contributed to the article and approved the submitted version.

## Funding

This study was supported by Grant 2013/15322-31, São Paulo Research Foundation (FAPESP).

## Conflict of Interest

The authors declare that the research was conducted in the absence of any commercial or financial relationships that could be construed as a potential conflict of interest.

## Publisher's Note

All claims expressed in this article are solely those of the authors and do not necessarily represent those of their affiliated organizations, or those of the publisher, the editors and the reviewers. Any product that may be evaluated in this article, or claim that may be made by its manufacturer, is not guaranteed or endorsed by the publisher.
